# Evidence for a Large Expansion and Subfunctionalization of Globin Genes in Sea Anemones

**DOI:** 10.1093/gbe/evy128

**Published:** 2018-06-27

**Authors:** Hayden L Smith, Ana Pavasovic, Joachim M Surm, Matthew J Phillips, Peter J Prentis

**Affiliations:** 1 School of Earth, Environmental and Biological Sciences, Queensland University of Technology, Brisbane, Queensland, Australia; 2 School of Biomedical Sciences, Queensland University of Technology, Brisbane, Queensland, Australia; 3 Institute for Future Environments, Queensland University of Technology, Brisbane, Queensland, Australia

**Keywords:** Cnidaria, gene duplication, hexacoordination, pentacoordination, phylogenetics

## Abstract

The globin gene superfamily has been well-characterized in vertebrates, however, there has been limited research in early-diverging lineages, such as phylum Cnidaria. This study aimed to identify globin genes in multiple cnidarian lineages, and use bioinformatic approaches to characterize the evolution, structure, and expression of these genes. Phylogenetic analyses and in silico protein predictions showed that all cnidarians have undergone an expansion of globin genes, which likely have a hexacoordinate protein structure. Our protein modeling has also revealed the possibility of a single pentacoordinate globin lineage in anthozoan species. Some cnidarian globin genes displayed tissue and development specific expression with very few orthologous genes similarly expressed across species. Our phylogenetic analyses also revealed that eumetazoan globin genes form a polyphyletic relationship with vertebrate globin genes. Overall, our analyses suggest that a Ngb-like and GbX-like gene were most likely present in the globin gene repertoire for the last common ancestor of eumetazoans. The identification of a large-scale expansion and subfunctionalization of globin genes in actiniarians provides an excellent starting point to further our understanding of the evolution and function of the globin gene superfamily in early-diverging lineages.

## Introduction

Globins are among the most studied gene superfamilies, and their evolution, structure, and expression is well-established in vertebrates (subphylum Vertebrata). There have been 11 globin protein subfamilies characterized in metazoans, which are the result of whole genome and single gene duplication events that have predominantly occurred among vertebrates (Hoffmann et al. 2012). Furthermore, some globin subfamilies have shown tissue and/or development specific expression (Burmester et al. 2000; Roesner et al. 2005; Hoogewijs et al. 2012; Koch et al. 2016). Phylogenetic analyses have demonstrated that either neuroglobin (Ngb), globin-X (GbX), or androglobin is the most ancient globin gene in vertebrates and metazoans in general (Burmester and Hankeln 2014). Of note, these three ancient globin proteins and cytoglobin have a hexacoordinate structural conformation, whereas, pentacoordination is observed in the seven other globin protein subfamilies (Burmester and Hankeln 2014). This variation in protein structure changes the binding position of the heme molecule and ligand binding affinities to various gaseous compounds (Dewilde et al. 2001; Nienhaus et al. 2004; Brunori et al. 2005; Fago et al. 2006; Jayaraman et al. 2011). These characteristics are well defined in vertebrates, however, this is not the case for metazoan lineages outside of vertebrates, especially for early-diverging lineages, such as cnidarians.

Globin-like genes have been identified in early-diverging phyla, including Ctenophora (Ryan et al. 2013), Porifera (Srivastava et al. 2010), Placozoa (Srivastava et al. 2008), and Cnidaria (Putnam et al. 2007; Chapman et al. 2010; Shinzato et al. 2011; Lechauve et al. 2013). Of these phyla, cnidarians are an ancient lineage, sister to superphylum Bilateria, possess a rudimentary nervous system and only two dermal layers (ectoderm and endoderm), and are reliant on diffusion to supply oxygen to working cells (Brusca and Brusca 2003; Technau and Steele 2011). Consequently, cnidarians are an excellent candidate group to study the early evolution, structure, and expression of globin genes and proteins, especially in comparison to the known characteristics of bilaterian globin genes. Two Ngb-like genes which displayed tissue specific expression have been identified in the hydroid *Clytia hemisphaerica* (phylum Cnidaria) (Lechauve et al. 2013). Outside of this study, very limited research has examined globin genes in phylum Cnidaria and hence we know little about the evolution, expression, and structure of these genes in early-diverging metazoans. The lack of knowledge surrounding globin genes outside of bilaterian species could be resolved by examining globin-like genes in phylum Cnidaria.

In this study, we used bioinformatic approaches to identify, characterize, and examine expression patterns of globin genes in phylum Cnidaria, specifically focusing on sea anemones (order Actiniaria). We analyzed published genomic data to identify globin-like genes across four classes of Cnidaria, Anthozoa (sea anemones, corals, and sea pens), Cubozoa (box jellyfish), Hydrozoa (hydras and hydroids), and Scyphozoa (true jellyfish), as well as sequenced genomes from phyla Porifera (sponges), Placozoa, and Ctenophora (comb jellies). We identified that cnidarian globin genes have undergone repeated rounds of duplication particularly in order Actiniaria, which have up to ten globin genes. Predictive protein modeling revealed possible structural variations in the heme pocket between different groups of actiniarian globin proteins, specifically the different gene clades that contained proteins with either pentacoordinate or hexacoordinate conformations. We also elucidated tissue and development specific patterns within the order Actiniaria; these two features are common in vertebrate systems.

## Materials and Methods

### Transcriptome Construction and Quality Checking

Transcriptome data sets generated with Illumina platforms were obtained from NCBI GenBank; *Acropora digitifera* (PRJNA309168; Mohamed et al. 2016), *Actinia tenebrosa* (SRX1604071), *Alatina alata* (SRX978662), *Anthopleura buddemeieri* (SRX1604661; van der Burg et al. 2016), *Aulactinia verata* (SRX1614867; van der Burg et al. 2016), *Aurelia aurita* (PRJNA252562; Brekhman et al. 2015), *Calliactis polypus* (SRX1614869; van der Burg et al. 2016), *Chironex fleckeri* (SRX891607), *Corrallium rubrum* (SRX675792; Pratlong et al. 2015), *Hydractinia polyclina* (SRX315374), *Nemanthus annamensis* (SRX1634628; van der Burg et al. 2016), *Protopalythoa variabilis* (SRX978667). Trinity de novo assembler software (v2.0.6) was used to assemble high quality reads (> Q30, < 1% ambiguities) into contiguous sequences (contigs) (Haas et al. 2013). Default settings were used with the addition of Trimmomatic to remove low quality reads and adaptors (Haas et al. 2013). Redundant and chimeric sequences present in the transcriptome were removed using CD-hit (v4.6.1) by clustering sequences with > 95% similarity into a single contig (Fu et al. 2012). The quality and completeness of the transcriptome assemblies were determined with CEGMA to report the presence of the 248 core eukaryotic genes (CEG) that were complete (> 70% alignment with CEG protein) (Parra et al. 2007) and BUSCO to report the presence of the 978 single-copy orthologs in metazoans that were complete (Simão et al. 2015). A CEGMA and BUSCO score of > 80% was considered high quality.

### Candidate Gene Identification

BLAST searches using vertebrate globin sequences against the genomes of *Nematostella vectensis* (Putnam et al. 2007)*, A. digitifera* (Shinzato et al. 2011)*, Hydra vulgaris* (Chapman et al. 2010)*, Trichoplax adhaerens* (Srivastava et al. 2008)*, Amphimedon queenslandica* (Srivastava et al. 2010), *and Mnemiopsis leidyi* (Ryan et al. 2013) were conducted. Potential globin gene sequences were extracted from genome scaffolds and transcripts.

Transcriptomes were annotated using the SwissProt database (Haas et al. 2013) within the Trinotate software package (v2.0.6) with an e-value stringency of 1e^−6^. A custom BLAST database was created using globin genes annotated in the *N. vectensis* and *H. vulgaris* genomes. Transcriptomes were locally blasted against this custom database to ensure any predicted proteins were contained in the globin gene candidate list. Candidate sequences were further scrutinized against the genome and transcriptome assemblies of the dinoflagellate, *Symbiodinium minutum*, using the Okinawa Institute of Science and Technology Graduate University’s Marine Genomics Unit genome browser (http://marinegenomics.oist.jp/symb/viewer/info?project_id=21). Sequences with an e-value <1e^−6^ were considered as potential dinoflagellate genes and subsequently removed from downstream analyses.

Candidate genes were assigned a custom nomenclature using the OrthoMCL database (http://orthomcl.org/orthomcl/) (Chen et al. 2006). Candidate genes were translated into protein sequences and queried against the OrthoMCL database to assign these genes to orthologous groups. They were assigned a custom nomenclature based on species and the best orthologous protein hit (supplementary table 1, Supplementary Material online).

### Candidate Gene Validation and Interrogation

Candidate sequences from *A. tenebrosa* and *Exaiptasia pallida* were validated using PCR amplification and Sanger sequencing. Primers were designed using the NCBI primer design tool (Ye et al. 2012) in order to amplify the entire open reading frame of the candidate globin genes (supplementary table 2, Supplementary Material online). PCR amplification of candidate genes was achieved using the MyFi2x Taq Polymerase Kit (BIOLINE); 12.5 µl MyFi2x polymerase master mix, 9.5 µl ddH_2_O, 1 µl 10 pmol Forward primer, 1 µl 10 pmol Reverse primer, 1 µl (20–50 ng) cDNA template. Sanger sequencing was completed using a modified BigDye Terminator v3.1 protocol (Applied Biosystems). Sanger sequences were aligned and mapped back to ORFs of the candidate gene they were designed from to validate assembly of candidate globin genes in these two species.

Validated candidate sequences from *E. pallida* were mapped back to the genome (Baumgarten et al. 2015) and the intron–exon structures were interrogated. Candidate genes were used as BLAST queries against the genome to identify the scaffolds they occurred on. Sequences were mapped back to these scaffolds using the Geneious software (v9), and intron–exon boundaries were determined using the typical GT…AG splicing rule.

### Phylogenetic Analysis

The distribution and diversification of globin-like genes was analyzed using Maximum Likelihood, and Bayesian Inference phylogenetic methods. Exonic nucleotide sequences comprising the globin protein domain (PFAM ID: PF00042) were aligned in MEGA (v6.06) (Tamura et al. 2013) using Muscle codon modeling (Edgar 2004). The best fit model test was conducted in MEGA (v6.06) for all phylogenetic trees. Subsequent phylogenetic analyses were conducted using IQ-TREE (http://iqtree.cibiv.univie.ac.at) (Trifinopoulos et al. 2016) and MrBayes (v3.2) (Ronquist et al. 2012). All phylogenies for nucleotide sequences were undertaken using the General Time Reversal model with gamma distribution and invariant sites. Maximum likelihood analyses were completed for 1,000 ultrafast bootstrap replications, using Codon F3x4 state frequency, ascertainment bias correction and 0.95 minimum correlation coefficient. Bayesian Inference analyses were completed for 10,000,000 MCMC generations, sampling every 1,000th generation, and used a default burn-in value of 25% (2,500 samples). The outgroup used for all phylogenetic trees was a *S. minutum* gene containing a single globin domain.

Outputs from IQ-TREE and MrBayes were further analyzed using topology tests in IQ-TREE (Trifinopoulos et al. 2016) and ancestral state reconstruction in MrBayes (Ronquist et al. 2012), respectively. Topology testing, with the same settings as above, was used to analyze constrained ML trees using the Approximately Unbiased test (Shimodaira 2002). Constrained trees forced cnidarian sequences into a clade with either Ngb or Ngb and GbX sequences. Ancestral state reconstructions were determined for three different classifications of cnidarian globin genes (globin-like, globin-X-like, and neuroglobin-like) using constraints for eumetazoan taxa, and either 1) cnidarian sequences with GbX sequences, or 2) GbX and Ngb with cnidarian sequences removed. All other globin genes were classified either as early diverging, hemoglobin, myoglobin, cytoglobin, or unknown vertebrate. Ancestral state probabilities were completed with default settings as per Ronquist et al. (2012), with the following changes: 2,000,000 MCMC generations, sampling every 2,000th generation, and diagnosis frequency every 50,000th generation.

### Protein Modeling Prediction

Validated candidate globin genes for *A. tenebrosa* and *E. pallida* were used to model predictive protein structures. Amino acid sequences were used in RaptorX (Källberg et al. 2012) to align against the Protein Data Bank with a stringency value of ≤1e^−3^. This relatively low value was used due to the lack of invertebrate globin-like protein structures available. Predictive models were subsequently loaded into the Chimera protein editor (Pettersen et al. 2004) to annotate and visualize protein structures, and manually align candidate cnidarian globin proteins for comparative analyses.

### Differential Gene Expression Analysis

Tissue and development specific transcriptome data sets generated with Illumina platforms were obtained from NCBI GenBank; *A. tenebrosa* (PRJNA350366) and *N. vectensis* (PRJEB13676; Babonis et al. 2016) for tissue data, *and N. vectensis* (PRJNA213177) and *E. pallida* (PRJNA261862; Baumgarten et al. 2015) for developmental data. Each individual data set was assembled with all raw reads combined into a single assembly as per the above *Transcriptome construction and quality checking* section. The Trinity RNA-seq software pipeline for determining differential gene expression was used for each of the assembled data sets (Haas et al. 2013). Raw reads were mapped back to the relevant combined assembly to obtain transcript abundance using the RSEM estimation method (Li and Dewey 2011) and the bowtie alignment method (Langmead et al. 2009). Principle components analysis was performed on the RSEM abundance count and normalized FPKM data outputs to ensure no batch effects were present. Differential expression was conducted using default settings in the Trinity RNA-seq software pipeline for the edgeR method (Likelihood Ratio Test) with a dispersion value of 0.1 (Robinson et al. 2010; Haas et al. 2013). Differentially expressed genes were considered significant provided they had a false discovery rate *P* value of <1e^−3^. Heatmaps were constructed in the Trinity RNA-seq software pipeline using default Perl to R sample correlation matrix settings for the normalized data and a log2-fold change centred on the mean, with minimum row and column expression values of 0 (Haas et al. 2013).

## Results

### Transcriptome Assembly and Candidate Gene Validation

All assembled transcriptomes were high quality based on their N50 values, and CEGMA and BUSCO completeness scores (supplementary table 3, Supplementary Material online). All transcriptomes had N50 values >1,000, with the exception of *A. aurita*. All transcriptomes had CEGMA and BUSCO scores > 80%, with the exception of *Chironex fleckeri*.

Analysis of genome sequences from early-diverging lineages (*N. vectensis, A. digitifera, H. vulgaris, T. adhaerens, A. queenslandica, and M. leidyi*) identified a total of 23 globin-like genes in the six different taxa examined (supplementary table 4 and supplementary fig. 1, Supplementary Material online). The three cnidarian species *N. vectensis, A. digitifera*, and *H. vulgaris* had nine, three and four globin-like genes, respectively, while *T. adhaerens* (Placozoa) had five globin-like genes and *M. leidyi* (Ctenophora) and *A. queenslandica* (Porifera) had one each.

Analysis of transcriptomic data identified a total of 74 globin-like genes from 15 different cnidarian taxa (supplementary table 4 and supplementary fig. 1, Supplementary Material online). Cnidarian species had up to ten globin-like genes. Compared with genome analyses, an additional globin-like gene was identified in the *N. vectensis* transcriptome (ortholog reference: N.vectensis_tadh6000210), and two additional candidate genes identified in the *A. digitifera* transcriptome (ortholog reference: A.digitifera_nvec42000019, A.digitifera_nvec76000030). The order Actiniaria had the greatest copy number with between five and ten globin-like genes identified in all species. In the medusozoa classes, *A. alata* and *C. fleckeri* (Cubozoa) each had one globin-like gene, *A. aurita* (Scyphozoa) had five globin-like genes, while *H. polyclina* (Hydrozoa) had four globin-like genes.

Seven globin-like genes in *A. tenebrosa* and nine globin-like genes in *E. pallida* were validated using Sanger sequencing with identity matches of ≥ 99.8% and ≥ 98.6%, respectively. A nonsynonymous mutation was observed at nucleotide position 139 for *A. tenebrosa* ortholog reference A.tenebrosa_nvec76000030, resulting in an amino acid change from Lysine (K) to Glutamic Acid (E). Synonymous and nonsynonymous mutations (between 0–5 and 0–2, respectively) were observed in all *E. pallida* candidate genes (supplementary table 5, Supplementary Material online) which likely reflects the different sampling locations, Saudi Arabia (NCBI accession number: PRJNA261862) versus Australia. The GbX membrane binding motif (MGC) (Blank et al. 2011) was identified in six *A. tenebrosa* globin-like proteins and seven *E. pallida* globin-like proteins (motif not observed in ortholog reference tadh6000210). The majority of candidate globin genes in sea anemones have the membrane binding motif, whereas, the majority of candidate globin genes in all other cnidarians analyzed lacked this motif. Additionally, a 2/3 intron–exon structure is present in all nine sequences of *E. pallida* (supplementary table 6, Supplementary Material online), with predicted intron start locations at helix positions B12.2 and G7.0, a pattern typical of metazoan globin genes.

### Evolutionary and Structural Analyses

**Fig. 1.**
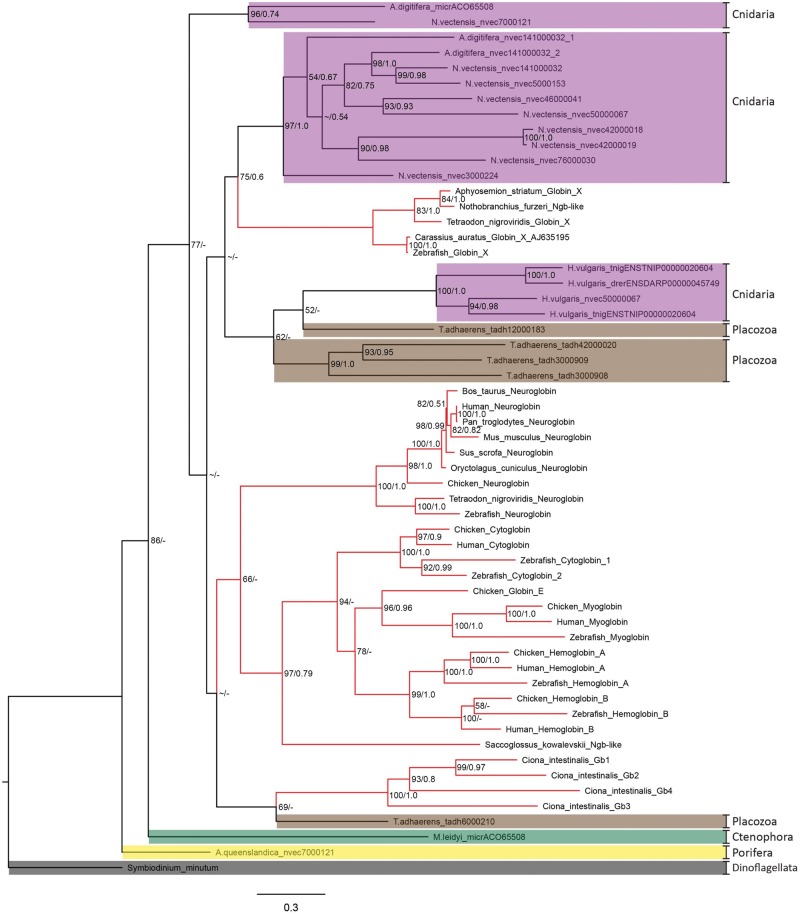
—Maximum likelihood (ML) phylogenetic tree of identified candidate cnidarian globin genes in genomes of cnidarian species, with ML bootstrap and Bayesian posterior probability support values. Model species representations of phyla Cnidaria, Ctenophora, Placozoa, and Porifera (highlighted in purple, green, brown, and yellow, respectively) with vertebrate globin genes highlighted with red branches, and the *Symbiodinium minutum* outgroup highlighted in gray. Support values shown as ML bootstrap support (0–100)/Bayesian posterior probabilities (0–1.0). Bootstrap values <50 and posterior probabilities <0.5 shown with a ∼ symbol and nodes not identical between each method shown with a - symbol.

**Fig. 2.**
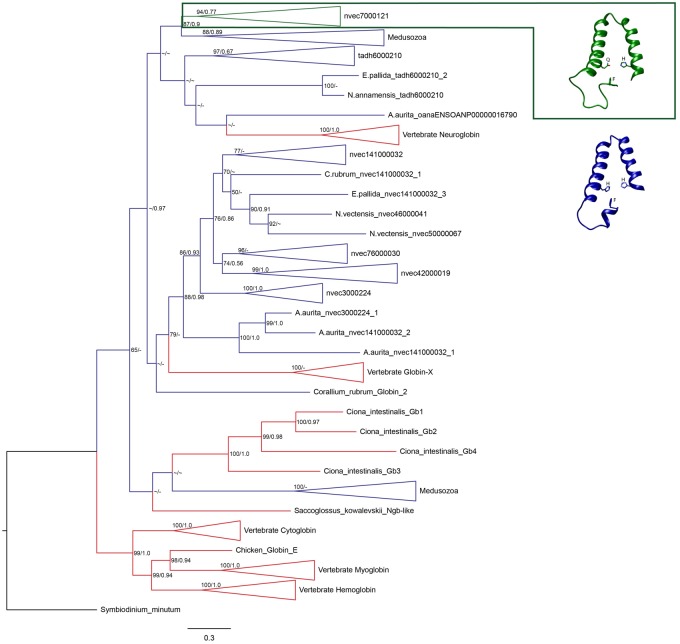
—Maximum likelihood (ML) phylogenetic tree of identified candidate cnidarian globin genes in transcriptomes of cnidarian species, with ML bootstrap and Bayesian posterior probability support values. Cnidarian pentacoordinate and hexacoordinate branches highlighted in green and blue, respectively, with vertebrate branches highlighted in red and the *Symbiodinium minutum* outgroup branch highlighted in black. Pentacoordinate cnidarian genes represented in ortholog reference nvec7000121 are associated with protein model highlighted in green. Hexacoordinate cnidarian genes are associated with protein model highlighted in blue. Support values shown as ML bootstrap support (0–100)/Bayesian posterior probabilities (0–1.0). Bootstrap values <50 and posterior probabilities <0.5 shown with a ∼ symbol and nodes not identical between each method shown with a - symbol. Collapsed clades represent sequences with the corresponding ortholog reference gene nomenclature as referenced in supplementary table 3, Supplementary Material online (expanded clades shown in supplementary fig. 2, Supplementary Material online).

Phylogenetic analysis of globin sequences derived from genome data (fig. 1) showed that the majority of anthozoan globin genes fall within their own clade and are sister to vertebrate GbX genes with moderate support values. However, other globin genes from early-diverging lineages are paraphyletic with or fall outside of vertebrate globin genes. Comparative phylogenetic analysis of transcriptome data (fig. 2) showed that the majority of cnidarian globin genes are monophyletic with vertebrate GbX (strong support) or Ngb (weak support). However, some cnidarian globin genes fall outside these clades, specifically ortholog reference nvec7000121 and medusozoan globin genes.

Topological and ancestral analyses of globin sequences derived from genome data revealed that figure 1 is an accurate representation of phylogenetic distribution. Approximately unbiased tests suggest that it is highly unlikely (*P* value < 0.07) that cnidarian globin genes are monophyletic with either Ngb or Ngb and GbX. However, ancestral state inferences revealed that Ngb and cnidarian globin genes are the favored ancestral state over GbX.

Alignment of cnidarian globin genes showed that three amino acid residues were highly conserved; Phenylalanine (F, CD1 position), Histidine/Glutamine (H/Q, E7 position), and Histidine (H, F8 position). Figures 1 and 2 revealed the E7 amino acid replacement between ortholog reference nvec7000121 (Q), and all other cnidarian sequences (H), likely changing these proteins to a pentacoordinate conformation from a hexacoordinate conformation. Interestingly, the presence of possible pentacoordinate globin proteins was only identified in the class Anthozoa, suggesting a unique role that has neither been characterized nor elucidated from any other class in phylum Cnidaria. Phylogenetic analysis revealed that predicted proteins with pentacoordinate confirmation have arisen once, but hexacoordinate predicted protein sequences have undergone an expansion in actiniarian species (fig. 2).

**Fig. 3.**
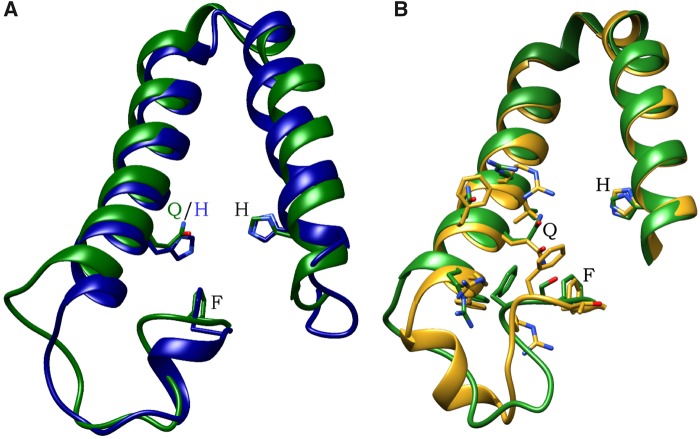
—Predictive cnidarian globin protein structure with heme pocket residues shown. (*A*) Structural variation of *Actinia tenebrosa* ortholog references A.tenebrosa_nvec7000121 (highlighted green) and A.tenebrosa_nvec42000019 (highlighted blue) with side chain residue structures for F (CD1 position; phenylalanine), Q/H (E7 position; distal glutamine/histidine), and H (F8 position; proximal histidine) shown. (*B*) Structural variation of *A. tenebrosa* ortholog reference A.tenebrosa_nvec7000121 (highlighted green) and *Exaiptasia pallida* ortholog reference E.pallida_nvec7000121 (highlighted gold) showing forward and reverse position of E7 residue Q, respectively, and with side chain residues surrounding E7 position shown.

Protein models for all validated sequences in *A. tenebrosa* and *E. pallida* showed similar conserved structures between these two species. Of note, models designated as pentacoordinate and hexacoordinate showed the amino acid change at the CD1 position (fig. 3*A*). Interestingly, the pentacoordinate sequences revealed a forward and reverse position for the distal Glutamine residue in *A. tenebrosa* and *E. pallida*, respectively (fig. 3*B*). The positions of the surrounding residues highlight the significance of steric hindrance on protein structure, especially for ligand binding to the heme group.

### Differential Gene Expression Analyses

Tissue and developmental data assemblies passed quality checking with the exception of two data sets from the *N. vectensis* development assembly. These data sets (NCBI Accession: SRX351436 and SRX351430) displayed batch effect outliers because of the different ribosomal RNA treatments used, and subsequently were excluded from downstream analysis. The development specific data set was further refined to include only the planula and adult stages to represent the same developmental stages from *E. pallida* and *N. vectensis*.

**Fig. 4.**
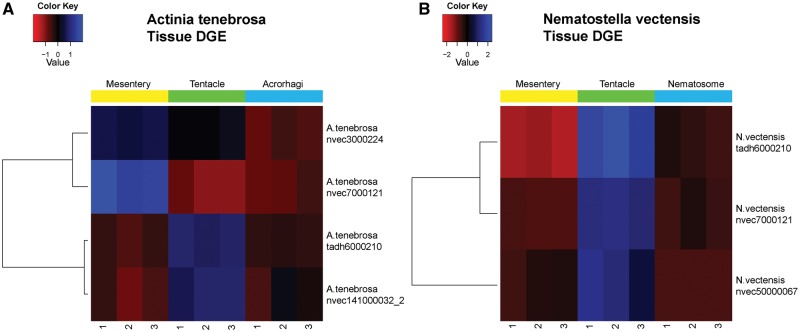
—Heatmap for tissue specific RNA-seq differential gene expression (DGE) analysis with three biological replicates for each tissue type. (*A*) Analysis of *Actinia tenebrosa* tissue types: acrorhagi, tentacle, and mesentery filament. (*B*) Analysis of *Nematostella vectensis* tissue types: nematosome, tentacle, and mesentery filament.

Tissue specific data revealed four and three cnidarian globin genes were differentially expressed in *A. tenebrosa* and *N. vectensis*, respectively (fig. 4). These globin genes were downregulated/upregulated in the acrorhagi of *A. tenebrosa* and the nematosome of *N. vectensis*. Two globin genes (ortholog references: tadh6000210, nvec141000032/nvec50000067) were upregulated in tentacle, in both *A. tenebrosa* and *N. vectensis*, while being downregulated in the mesenteric filament. Ortholog reference nvec7000121 was upregulated in mesenteric filament of *A. tenebrosa*, whereas, it was upregulated in tentacle of *N. vectensis*.

**Fig. 5.**
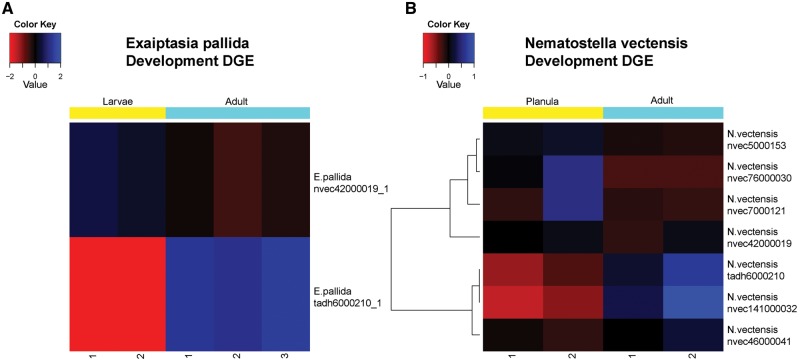
—Heatmap for development specific RNA-seq differential gene expression (DGE) analysis with two biological replicates for each tissue type. (*A*) Analysis of *Exaiptasia pallida* developmental stages: immature (larvae) and mature (adult), with three biological replicates for adult stage only. (*B*) Analysis of *Nematostella vectensis* developmental stages: immature (planula) and mature (adult).

Development specific data revealed seven and two cnidarian globin genes were differentially expressed in *N. vectensis* and *E. pallida*, respectively (fig. 5). *E. pallida* has one globin gene upregulated at the immature stage, and the other upregulated at the mature stage. The globin sequence from the *N. vectensis* transcriptome (ortholog reference: tadh6000210), also found in the tissue specific data, was present in the development specific transcriptomic data. This ortholog was upregulated in both *E. pallida* and *N. vectensis* at the mature stage. Furthermore, there is a difference in expression pattern for cnidarian globin genes that cluster in the same clade observed in figure 2 (ortholog references: nvec5000153 and nvec141000032).

The tissue and developmental gene expression analyses of *N. vectensis* revealed that eight of the ten different orthologs were differentially expressed in one data set. Interestingly, only a single cnidarian globin gene (ortholog reference tadh6000210) was upregulated in both data sets, and this globin gene clusters closely with vertebrate Ngb (fig. 2).

## Discussion

The phylogenetic position of globin genes in early-diverging lineages is currently unclear, specifically the theorized monophyly with vertebrate Ngb, and their relationship to vertebrate globin genes in general. Dröge et al. (2012) have shown a single globin gene in *T. adhaerens* to be orthologous with Ngb and another two globin genes that cluster closely with Ngb. Whereas, Lechauve et al. (2013) have shown globin genes from phyla Cnidaria, Placozoa, and Porifera form a polyphyletic relationship with globin genes from vertebrate taxa. Our data supports the findings of Lechauve et al. (2013) that globin genes from early-diverging taxa do not form a single clade with vertebrate Ngb genes. In fact, most cnidarian globin genes form a moderately well-supported clade with vertebrate GbX, with some forming another clade containing vertebrate Ngb. The close relationship observed between many cnidarian globin genes and vertebrate GbX is supported by the presence of the membrane binding motif exclusive to GbX proteins in vertebrates. These results indicate that both a Ngb-like and GbX-like gene may have been present in the common ancestor of cnidarians and bilaterians. Our analyses provide a better understanding of the evolution of globin genes in early-diverging metazoans, however, they also highlight the need to resolve the evolutionary history of this ubiquitous gene superfamily.

An expansion of globin genes has been identified in various metazoan lineages, such as myoglobin in lungfish (Koch et al. 2016) and nerve globins in nematodes (Hoogewijs et al. 2008), but there have been limited studies of globin genes in early-diverging taxa. Our analyses of gene copy number and differential gene expression patterns suggests that large-scale duplication of cnidarian globin genes was followed by the subfunctionalization of gene expression in actiniarian species. This is similar to observations of lungfish myoglobin genes and nematode nerve globin genes that indicate subfunctionalization has followed multiple duplication events in these lineages (Hoogewijs et al. 2008; Koch et al. 2016). These studies combined with our data suggest subfunctionalization of gene expression after large-scale copy number increases are common for the globin gene superfamily.

Expression patterns of globin genes in actiniarians revealed some copies displayed tissue and development specific expression. The upregulation of tentacle specific globin genes suggests that they may have an alternative function to oxygen transport. The tentacles of actiniarians are in direct contact with the surrounding water and are likely to be constantly diffusing gaseous compounds with the surrounding environment. An alternative role for the globin genes upregulated in tentacles may be to provide cellular energy to replenish the dense concentration of stinging and attachment cells in the tentacle. In particular the continued replacement of cnidom, such as nematocysts and spirocysts, makes an oxygen storage role for mitochondrial ATP production and reactive oxygen species detoxification a possible function for these cnidarian globin genes, a role similar to vertebrate Ngb (Bentmann et al. 2005; Fordel et al. 2007). Furthermore, we found that ortholog reference tadh6000210, which clusters closely with vertebrate Ngb, was upregulated in the tentacle of *A. tenebrosa* and *N. vectensis* and the predicted protein encoded by this gene lacks the membrane binding motif found in vertebrate GbX. Consequently, this cnidarian globin gene represents the most likely candidate for an oxygen storage role and further highlights the potential subfunctionalization of globin genes in cnidarians.

The pentacoordinate and hexacoordinate conformations in vertebrate globin proteins have different affinities for ligands, and it is parsimonious that cnidarian globin proteins would exhibit similar ligand affinities under these different conformations. Ligands other than oxygen can be toxic at high concentrations, such as, nitric oxide and carbon monoxide. These compounds can be bound to the heme pocket of globin proteins, and this subsequently reduces the deleterious impact of these molecules on an organism (Dewilde et al. 2001; Brunori et al. 2005; Fago et al. 2006; Azarov et al. 2016). Recent studies examining carbon monoxide poisoning (Azarov et al. 2016) and nitrite reduction (Tejero et al. 2015) showed that pentacoordinate H64Q Ngb was a more effective ligand trap for toxic compounds than hexacoordinate H64 Ngb. Consequently, our modeling predictions of a single pentacoordinate cnidarian globin protein found exclusively in class anthozoa suggests a possible detoxification function for this protein.

We have identified a broad expansion of globin genes in phylum Cnidaria resulting from repeated rounds of gene duplication. In actiniarians, this expansion was followed by subfunctionalization that has resulted in tissue and developmental specific expression patterns, with possible structural/functional variation following one of these duplication events. Of particular interest is the single pentacoordinate clade that is restricted to class Anthozoa, which likely has a detoxification role. The identification of a large-scale expansion and subfunctionalization of cnidarian globin genes in actiniarians provides an excellent starting point to further our understanding of the evolution and function of the globin gene superfamily outside of phylum Chordata.

## Supplementary Material

Supplementary data are available at *Genome Biology and Evolution* online.

## Supplementary Material

Supplementary Data
