# Supplementary material for: Evidence for a Large Expansion and Subfunctionalization of Globin Genes in Sea Anemones
Source: Genome Biol Evol. 2018 Jun 27;10(8):1892–901. doi: 10.1093/gbe/evy128 (PMC6077788; doi:10.1093/gbe/evy128)

Supplementary Table 1. Output from OrthoMCL for candidate globin genes, with individual gene nomenclature used for all downstream analyses.

Candidate Gene Nomenclature	OrthoMCL Group	Genome Reference ID	E-value	%Ident	%Match
A.alata_oanaENSOANP00000016790	OG5_132086	oana|ENSOANP00000016790	$2e^{-20}$	33	96
A.aurita_nvec3000224_1	OG5_132086	nvec|fgenesh1_pg.scaffold_3000224	$1e^{-25}$	34	99
A.aurita_nvec141000032_1	OG5_132086	nvec|fgenesh1_pg.scaffold_141000032	$6e^{-16}$	31	91
A.aurita_nvec141000032_2	OG5_132086	nvec|fgenesh1_pg.scaffold_141000032	$2e^{-25}$	34	99
A.aurita_nvec3000224_2	OG5_132086	nvec|fgenesh1_pg.scaffold_3000224	$4e^{-18}$	29	99
A.aurita_oanaENSOANP00000016790	OG5_132086	oana|ENSOANP00000016790	$9e^{-17}$	33	83
A.buddemeieri_nvec5000153	OG5_132086	nvec|fgenesh1_pg.scaffold_5000153	$9e^{-66}$	60	99
A.buddemeieri_tadh6000210	OG5_132086	tadh|fgeneshTA2_pg.C_scaffold_6000210	$2e^{-28}$	40	92
A.buddemeieri_nvec7000121	OG5_146786	nvec|fgenesh1_pg.scaffold_7000121	$5e^{-35}$	39	99
A.buddemeieri_nvec141000032	OG5_132086	nvec|fgenesh1_pg.scaffold_141000032	$6e^{-67}$	69	99
A.buddemeieri_nvec76000030	OG5_132086	nvec|fgenesh1_pg.scaffold_76000030	$3e^{-42}$	47	100
A.buddemeieri_nvec3000224	OG5_132086	nvec|fgenesh1_pg.scaffold_3000224	$6e^{-62}$	56	100
A.buddemeieri_nvec42000019	OG5_132086	nvec|fgenesh1_pg.scaffold_42000019	$2e^{-63}$	61	99
A.digitifera_micrACO65508	OG5_132086	micr|ACO65508	$8e^{-24}$	41	97
A.digitifera_nvec141000032_1	OG5_132086	nvec|fgenesh1_pg.scaffold_141000032	$1e^{-34}$	43	98
A.digitifera_nvec141000032_2	OG5_132086	nvec|fgenesh1_pg.scaffold_141000032	$1e^{-65}$	67	100
A.digitifera_nvec76000030	OG5_132086	nvec|fgenesh1_pg.scaffold_76000030	$2e^{-36}$	42	99
A.digitifera_nvec42000019	OG5_132086	nvec|fgenesh1_pg.scaffold_42000019	$8e^{-24}$	44	98
A.queenslandica_nvec7000121	OG5_146786	nvec|fgenesh1_pg.scaffold_7000121	$3e^{-18}$	34	97
A.tenebrosa_nvec42000019	OG5_132086	nvec|fgenesh1_pg.scaffold_42000019	$1e^{-65}$	64	99
A.tenebrosa_nvec7000121	OG5_146786	nvec|fgenesh1_pg.scaffold_7000121	$2e^{-34}$	40	97
A.tenebrosa_tadh6000210	OG5_132086	tadh|fgeneshTA2_pg.C_scaffold_6000210	$8e^{-29}$	42	92
A.tenebrosa_nvec76000030	OG5_132086	nvec|fgenesh1_pg.scaffold_76000030	$3e^{-42}$	48	98
A.tenebrosa_nvec3000224	OG5_132086	nvec|fgenesh1_pg.scaffold_3000224	$4e^{-62}$	57	100
A.tenebrosa_nvec141000032_1	OG5_132086	nvec|fgenesh1_pg.scaffold_141000032	$6e^{-65}$	68	99
A.tenebrosa_nvec141000032_2	OG5_132086	nvec|fgenesh1_pg.scaffold_141000032	$3e^{-67}$	70	99
A.veratra_nvec7000121	OG5_146786	nvec|fgenesh1_pg.scaffold_7000121	$1e^{-31}$	37	97
A.veratra_nvec141000032_1	OG5_132086	nvec|fgenesh1_pg.scaffold_141000032	$9e^{-67}$	71	99
A.veratra_nvec141000032_2	OG5_132086	nvec|fgenesh1_pg.scaffold_141000032	$1e^{-65}$	67	98

A.veratra_nvec76000030	OG5_132086	nvec|fgenesh1_pg.scaffold_76000030	$4e^{-47}$	52	100
A.veratra_nvec3000224	OG5_132086	nvec|fgenesh1_pg.scaffold_3000224	$4e^{-62}$	58	99
A.veratra_nvec42000019	OG5_132086	nvec|fgenesh1_pg.scaffold_42000019	$3e^{-65}$	63	99
A.veratra_tadh6000210	OG5_132086	tadh|fgeneshTA2_pg.C_scaffold_6000210	$3e^{-29}$	40	92
C.fleckeri_nvec7000121	OG5_146786	nvec|fgenesh1_pg.scaffold_7000121	$7e^{-22}$	34	77
C.polypus_nvec141000032	OG5_132086	nvec|fgenesh1_pg.scaffold_141000032	$1e^{-65}$	71	99
C.polypus_tadh6000210	OG5_132086	tadh|fgeneshTA2_pg.C_scaffold_6000210	$1e^{-25}$	40	92
C.polypus_nvec42000019	OG5_132086	nvec|fgenesh1_pg.scaffold_42000019	$4e^{-61}$	60	98
C.polypus_nvec7000121	OG5_146786	nvec|fgenesh1_pg.scaffold_7000121	$1e^{-29}$	37	99
C.polypus_nvec76000030	OG5_132086	nvec|fgenesh1_pg.scaffold_76000030	$2e^{-35}$	49	100
C.rubrum_nvec141000032_1	OG5_132086	nvec|fgenesh1_pg.scaffold_141000032	$2e^{-39}$	46	99
C.rubrum_nvec141000032_2	OG5_132086	nvec|fgenesh1_pg.scaffold_141000032	$1e^{-19}$	28	99
C.rubrum_nvec7000121	OG5_146786	nvec|fgenesh1_pg.scaffold_7000121	$1e^{-25}$	35	81
E.pallida_nvec42000019_1	OG5_132086	nvec|fgenesh1_pg.scaffold_42000019	$4e^{-47}$	51	97
E.pallida_nvec3000224	OG5_132086	nvec|fgenesh1_pg.scaffold_3000224	$5e^{-63}$	57	99
E.pallida_tadh6000210_1	OG5_132086	tadh|fgeneshTA2_pg.C_scaffold_6000210	$9e^{-28}$	42	90
E.pallida_nvec141000032_1	OG5_132086	nvec|fgenesh1_pg.scaffold_141000032	$2e^{-67}$	73	99
E.pallida_nvec7000121	OG5_146786	nvec|fgenesh1_pg.scaffold_7000121	$2e^{-30}$	36	98
E.pallida_nvec141000032_2	OG5_132086	nvec|fgenesh1_pg.scaffold_141000032	$5e^{-66}$	71	99
E.pallida_tadh6000210_2	OG5_132086	tadh|fgeneshTA2_pg.C_scaffold_6000210	$9e^{-24}$	40	90
E.pallida_nvec141000032_3	OG5_132086	nvec|fgenesh1_pg.scaffold_141000032	$5e^{-38}$	44	99
E.pallida_nvec42000019_2	OG5_132086	nvec|fgenesh1_pg.scaffold_42000019	$2e^{-62}$	65	99
H.polyclina_tadh6000210	OG5_132086	tadh|fgeneshTA2_pg.C_scaffold_6000210	$3e^{-10}$	26	87
H.polyclina_mdomENSODP00000006771	OG5_132086	mdom|ENSMODP00000006771	$3e^{-8}$	22	92
H.polyclina_phumPHUM323880	OG5_132086	phum|PHUM323880	$4e^{-8}$	25	72
H.polyclina_trubENSTRU00000033639	OG5_132086	trub|ENSTRUP00000033639	$8e^{-9}$	23	85
H.vulgaris_tnigENSTNIP00000020604	OG5_132086	tnig|ENSTNIP00000020604	$2e^{-13}$	27	70
H.vulgaris_drerENSDARP00000045749	OG5_132086	drer|ENSDARP00000045749	$2e^{-13}$	27	81
H.vulgaris_nvec50000067	OG5_132086	nvec|fgenesh1_pg.scaffold_50000067	$1e^{-11}$	25	84
H.vulgaris_tnigENSTNIP00000020604	OG5_132086	tnig|ENSTNIP00000020604	$1e^{-13}$	29	71
M.leidyi_micrACO65508	OG5_132086	micr|ACO65508	$6e^{-10}$	31	85
N.annamensis_nvec141000032	OG5_132086	nvec|fgenesh1_pg.scaffold_141000032	$6e^{-62}$	64	99
N.annamensis_tadh6000210_1	OG5_132086	tadh|fgeneshTA2_pg.C_scaffold_6000210	$1e^{-28}$	44	92

N.annamensis_tadh6000210_2	OG5_132086	tadh|fgeneshTA2_pg.C_scaffold_6000210	$3e^{-24}$	40	100
N.annamensis_nvec7000121	OG5_146786	nvec|fgenesh1_pg.scaffold_7000121	$2e^{-30}$	36	99
N.annamensis_nvec42000019	OG5_132086	nvec|fgenesh1_pg.scaffold_42000019	$2e^{-55}$	55	99
N.annamensis_nvec76000030	OG5_132086	nvec|fgenesh1_pg.scaffold_76000030	$3e^{-43}$	48	100
N.annamensis_nvec141000032_1	OG5_132086	nvec|fgenesh1_pg.scaffold_141000032	$3e^{-66}$	71	99
N.annamensis_nvec141000032_2	OG5_132086	nvec|fgenesh1_pg.scaffold_141000032	$2e^{-67}$	72	99
N.vectensis_nvec3000224	OG5_132086	nvec|fgenesh1_pg.scaffold_3000224	$1e^{-106}$	100	100
N.vectensis_nvec141000032	OG5_132086	nvec|fgenesh1_pg.scaffold_141000032	$6e^{-98}$	100	100
N.vectensis_nvec46000041	OG5_132086	nvec|fgenesh1_pg.scaffold_46000041	$2e^{-77}$	100	100
N.vectensis_nvec7000121	OG5_146786	nvec|fgenesh1_pg.scaffold_7000121	$1e^{-114}$	100	100
N.vectensis_nvec42000018	OG5_132086	nvec|fgenesh1_pg.scaffold_42000018	$1e^{-138}$	100	100
N.vectensis_nvec42000019	OG5_132086	nvec|fgenesh1_pg.scaffold_42000019	$1e^{-104}$	100	100
N.vectensis_nvec76000030	OG5_132086	nvec|fgenesh1_pg.scaffold_76000030	$1e^{-107}$	100	100
N.vectensis_nvec5000153	OG5_132086	nvec|fgenesh1_pg.scaffold_5000153	$1e^{-124}$	100	100
N.vectensis_nvec50000067	OG5_132086	nvec|fgenesh1_pg.scaffold_50000067	$1e^{-105}$	100	100
N.vectensis_tadh6000210	OG5_132086	tadh|fgeneshTA2_pg.C_scaffold_6000210	$1e^{-23}$	41	96
P.variabilis_tadh6000210	OG5_132086	tadh|fgeneshTA2_pg.C_scaffold_6000210	$3e^{-25}$	39	86
P.variabilis_micrACO65508	OG5_132086	micr|ACO65508	$2e^{-18}$	38	88
T.adhaerens_tadh12000183	OG5_174830	tadh|fgeneshTA2_pg.C_scaffold_12000183	$2e^{-64}$	100	100
T.adhaerens_tadh42000020	OG5_211503	tadh|fgeneshTA2_pg.C_scaffold_42000020	$1e^{-103}$	100	100
T.adhaerens_tadh6000210	OG5_132086	tadh|fgeneshTA2_pg.C_scaffold_6000210	$7e^{-91}$	100	100
T.adhaerens_tadh3000908	OG5_173496	tadh|fgeneshTA2_pg.C_scaffold_3000908	$8e^{-75}$	100	100
T.adhaerens_tadh3000909	OG5_211503	tadh|fgeneshTA2_pg.C_scaffold_3000909	$3e^{-81}$	100	100

Supplementary Table 2: Primer sequences and estimated gene sequence length for candidate globin genes in *A. tenebrosa* and *E. pallida*. Candidate gene nomenclature referenced from OrthoMCL results detailed in Supplementary Table 3.

**Candidate Gene ID**	**NCBI Accession Number**	**Forward Primer Sequence**	**Reverse Primer Sequence**	**Estimated Gene Length**
A.tenebrosa_nvec141000032_1	KY810202	TTTTTCCGTCTCGAAGATA	CAAAGTGTACACCCTCTTC	579
A.tenebrosa_nvec141000032_2	KY810203	AAACCAAGATCGACCAGTT	TACAGATCTAGACCAGGAAAG	588
A.tenebrosa_nvec3000224	KY810201	TCTTTTCAAGTTTTCCTAGCC	GGCAAGACTTTTCCAGTTTA	609
A.tenebrosa_nvec42000019	KY810197	GAGTTAAGAATTCAAGAGGC	GCTGTTCACACAGATATAAAGA	640
A.tenebrosa_nvec7000121	KY810198	AGTTTTCTTGCTCTGTTCATC	CATGCGCATCACTGTTTG	577
A.tenebrosa_nvec76000030	KY810200	CACTGCTTAAAGTCCTCATTAT	CCTGTGCGTTCTCATGTA	604
A.tenebrosa_tadh6000210	KY810199	TGATGTCCAAAATACTGATGC	CCCTTGTCGATTGATAAAGTAT	648
E.pallida_nvec141000032_1	KY810207	TCCGACTAGGCGAAATTAAA	GTTCTTTATTCATGTTTGATGTG	582
E.pallida_nvec141000032_2	KY810209	TATACAAAGAAATCCTCAAGAGA	TTAGGTGGTCGATAGTGATG	564
E.pallida_nvec141000032_3	KY810211	CCTGGTTTGCCATATTGATTG	AAGATTCTTACATATGACAAGTGG	614
E.pallida_nvec3000224	KY810205	CTGATAGAGAAGTGACGAGAT	CGATACCGCTGAACATCAAT	580
E.pallida_nvec42000019_1	KY810204	ACCAACAATCTTCATTGAACT	TAGCCATAGATTTTACGTGGA	610
E.pallida_nvec42000019_2	KY810212	TTAATTTGAAGTCTTTCGTGAAG	AATTAGACTTTGGCTTTGAGC	590
E.pallida_nvec7000121	KY810208	TAAAATCGTTCACACATCGTT	GCTATTCGTACGAGAATGAAA	620
E.pallida_tadh6000210_1	KY810206	TAGGTGTACTGGGAATTTGAT	GACAGTAGGTAAAGCAAGAAG	544
E.pallida_tadh6000210_2	KY810210	TGAAGCAATAAGCAGTTCCC	CTAAAAAGAGATGTGATTGGCT	555

Supplementary Table 3: Trinity *De novo* assembled transcriptome statistics for quality check analysis. Abbreviations: n/a, Not Applicable.

Genus	Species	Accession Number	N50	No. Genes	No. Transcripts	CEGMA Score	BUSCO %	Accession Citation
Actinia	*tenebrosa*	SRX1604071	1,995	92,938	114,252	239	97.0	Van Der Burg et al. 2016
Actinia	*tenebrosa*	PRJNA350366	1,256	165,401	221,845	241	97.7	n/a
Acropora	*digitifera*	PRJNA309168	1,160	101,721	133,920	236	93.8	Mohamed et al. 2016
Alatina	*alata*	SRX978662	1,044	121,034	141,973	231	91.4	n/a
Anthopleura	*buddemeieri*	SRX1604661	1,034	150,702	212,774	220	94.6	Van Der Burg et al. 2016
Aulactinia	*verata*	SRX1614867	1,333	132,909	174,203	237	97.2	Van Der Burg et al. 2016
Aurelia	*aurita*	PRJNA252562	932	99,240	132,259	236	97.1	Brekhman et al 2015
Calliactis	*polypus*	SRX1614869	1,516	118,290	146,659	236	96.9	Van Der Burg et al. 2016
Chironex	*fleckeri*	SRX891607	1,377	46,983	51,149	200	74.7	n/a
Corrallium	*rubrum*	SRX675792	n/a	n/a	n/a	244	97.4	Pratlong et al. 2015
Exaiptasia	*pallida*	PRJNA261862	1,449	163,275	192,450	245	97.4	Baumgarten et al. 2015
Hydractinia	*polyclina*	SRX315374	1,300	135,939	159,235	242	95.8	n/a
Nemanthus	*annamensis*	SRX1634628	1,699	72,505	88,325	242	97.3	Van Der Burg et al. 2016
Nematostella	*vectensis*	PRJEB13676	1,033	301,047	369,434	232	86.8	Babonis et al. 2016
Nematostella	*vectensis*	PRJNA213177	1,255	133,272	153,212	222	96.5	n/a
Protopalythoa	*variabilis*	SRX978667	1,094	118,609	131,993	222	88.9	n/a

Supplementary Table 4. Results of data interrogation for genome and transcriptome datasets. Details represent additional information for individual candidate globin genes. Candidate gene nomenclature referenced from OrthoMCL results detailed in Supplementary Table 3.

Species and Candidate Count	Dataset	Accession Number	Candidate ID	Details
**Cnidaria**				
Acropora digitifera (3)	Genome	GCA_000222465.2	A.digitifera_micrACO65508	
	Genome	GCA_000222465.2	A.digitifera_nvec141000032_1	
	Genome	GCA_000222465.2	A.digitifera_nvec141000032_2	
Hydra vulgaris (4)	Genome	XM_012702974	H.vulgaris_drerENSDARP00000045749	
	Genome	XM_012711718	H.vulgaris_nvec50000067	
	Genome	XM_004209707	H.vulgaris_tnigENSTNIP00000020604_1	
	Genome	XM_004206290	H.vulgaris_tnigENSTNIP00000020604_2	
Nematostella vectensis (9)	Genome	XM_001629427	N.vectensis_nvec141000032	
	Genome	XM_001641595	N.vectensis_nvec3000224	
	Genome	XM_001636028	N.vectensis_nvec42000018	
	Genome	XM_001636029	N.vectensis_nvec42000019	
	Genome	XM_001635585	N.vectensis_nvec46000041	
	Genome	XM_001635260	N.vectensis_nvec50000067	
	Genome	XM_001640935	N.vectensis_nvec5000153	
	Genome	XM_001640512	N.vectensis_nvec7000121	
	Genome	XM_001633077	N.vectensis_nvec76000030	
Acropora digitifera (5)	Transcriptome	PRJNA309168	A.digitifera_micrACO65508	
	Transcriptome	PRJNA309168	A.digitifera_nvec141000032_1	
	Transcriptome	PRJNA309168	A.digitifera_nvec141000032_2	
	Transcriptome	PRJNA309168	A.digitifera_nvec42000019	Incomplete ORF; Full globin domain
	Transcriptome	PRJNA309168	A.digitifera_nvec76000030	
Actinia tenebrosa (7)	Transcriptome	SRX1604071	A.tenebrosa_nvec141000032_1	
	Transcriptome	SRX1604071	A.tenebrosa_nvec141000032_2	
	Transcriptome	SRX1604071	A.tenebrosa_nvec3000224	

	Transcriptome	SRX1604071	A.tenebrosa_nvec42000019	
	Transcriptome	SRX1604071	A.tenebrosa_nvec7000121	
	Transcriptome	SRX1604071	A.tenebrosa_nvec76000030	
	Transcriptome	SRX1604071	A.tenebrosa_tadh6000210	
Alatina alata (1)	Transcriptome	SRX978662	A.alata_oanaENSOANP00000016790	Tentacle only transcriptome
Anthopleura buddemeieri (7)	Transcriptome	SRX1604661	A.buddemeieri_nvec141000032	
	Transcriptome	SRX1604661	A.buddemeieri_nvec3000224	
	Transcriptome	SRX1604661	A.buddemeieri_nvec42000019	
	Transcriptome	SRX1604661	A.buddemeieri_nvec5000153	
	Transcriptome	SRX1604661	A.buddemeieri_nvec7000121	
	Transcriptome	SRX1604661	A.buddemeieri_nvec76000030	
	Transcriptome	SRX1604661	A.buddemeieri_tadh6000210	
Aulactinia veratra (7)	Transcriptome	SRX1614867	A.veratra_nvec141000032_1	
	Transcriptome	SRX1614867	A.veratra_nvec141000032_2	
	Transcriptome	SRX1614867	A.veratra_nvec3000224	
	Transcriptome	SRX1614867	A.veratra_nvec42000019	
	Transcriptome	SRX1614867	A.veratra_nvec7000121	
	Transcriptome	SRX1614867	A.veratra_nvec76000030	
	Transcriptome	SRX1614867	A.veratra_tadh6000210	
Aurelia aurita (5)	Transcriptome	PRJNA252562	A.aurita_nvec141000032_1	
	Transcriptome	PRJNA252562	A.aurita_nvec141000032_2	
	Transcriptome	PRJNA252562	A.aurita_nvec3000224_1	
	Transcriptome	PRJNA252562	A.aurita_nvec3000224_2	
	Transcriptome	PRJNA252562	A.aurita_oanaENSOANP00000016790	
Calliactis polypus (5)	Transcriptome	SRX1614869	C.polypus_nvec141000032	
	Transcriptome	SRX1614869	C.polypus_nvec42000019	
	Transcriptome	SRX1614869	C.polypus_nvec7000121	
	Transcriptome	SRX1614869	C.polypus_nvec76000030	Incomplete ORF; Full globin domain
	Transcriptome	SRX1614869	C.polypus_tadh6000210	
Chironex fleckeri (1)	Transcriptome	SRX891607	C.fleckeri_nvec7000121	Tentacle only transcriptome
Corallium rubrum (3)	Transcriptome	SRX675792	C.rubrum_nvec141000032_1	
	Transcriptome	SRX675792	C.rubrum_nvec141000032_2	

	Transcriptome	SRX675792	C.rubrum_nvec7000121
Exaiptasia pallida (9)	Transcriptome	PRJNA261862	E.pallida_nvec141000032_1
	Transcriptome	PRJNA261862	E.pallida_nvec141000032_2
	Transcriptome	PRJNA261862	E.pallida_nvec141000032_3
	Transcriptome	PRJNA261862	E.pallida_nvec3000224
	Transcriptome	PRJNA261862	E.pallida_nvec42000019_1
	Transcriptome	PRJNA261862	E.pallida_nvec42000019_2
	Transcriptome	PRJNA261862	E.pallida_nvec7000121
	Transcriptome	PRJNA261862	E.pallida_tadh6000210_1
	Transcriptome	PRJNA261862	E.pallida_tadh6000210_2
Hydractinia polyclina (4)	Transcriptome	SRX315374	H.polyclina_mdomENSODP00000006771
	Transcriptome	SRX315374	H.polyclina_phumPHUM323880
	Transcriptome	SRX315374	H.polyclina_tadh6000210
	Transcriptome	SRX315374	H.polyclina_trubENSTRU00000033639
Nemanthus annamensis (8)	Transcriptome	SRX1634628	N.annamensis_nvec141000032
	Transcriptome	SRX1634628	N.annamensis_nvec141000032
	Transcriptome	SRX1634628	N.annamensis_nvec141000032
	Transcriptome	SRX1634628	N.annamensis_nvec42000019
	Transcriptome	SRX1634628	N.annamensis_nvec7000121
	Transcriptome	SRX1634628	N.annamensis_nvec76000030
	Transcriptome	SRX1634628	N.annamensis_tadh6000210
	Transcriptome	SRX1634628	N.annamensis_tadh6000210
Nematostella vectensis (10)	Transcriptome	PRJNA213177	N.vectensis_nvec141000032
	Transcriptome	PRJNA213177	N.vectensis_nvec3000224
	Transcriptome	PRJNA213177	N.vectensis_nvec42000018
	Transcriptome	PRJNA213177	N.vectensis_nvec42000019
	Transcriptome	PRJNA213177	N.vectensis_nvec46000041
	Transcriptome	PRJNA213177	N.vectensis_nvec50000067
	Transcriptome	PRJNA213177	N.vectensis_nvec5000153
	Transcriptome	PRJNA213177	N.vectensis_nvec7000121
	Transcriptome	PRJNA213177	N.vectensis_nvec76000030
	Transcriptome	PRJNA213177	N.vectensis_tadh6000210
Protopalythoa variabilis (2)	Transcriptome	SRX978667	P.variabilis_micrACO65508

	Transcriptome	SRX978667	P.variabilis_tadh6000210
**Ctenophora**			
Mnemiopsis leidyi (1)	Genome	GCA_000226015.1	M.leidyi_micrACO65508
**Placozoa**			
Trichoplax adhaerens (5)	Genome	GCA_000150275.1	T.adhaerens_tadh3000908
	Genome	GCA_000150275.1	T.adhaerens_tadh3000909
	Genome	GCA_000150275.1	T.adhaerens_tadh6000210
	Genome	GCA_000150275.1	T.adhaerens_tadh42000020
	Genome	GCA_000150275.1	T.adhaerens_tadh12000183
**Porifera**			
Amphimedon queenslandica (1)	Genome	GCA_000090795.1	A.queenslandica_nvec7000121

Supplementary Table 5: Synonymous and nonsynonymous mutations identified in validated transcriptome contigs for *E. pallida* species. Abbreviations: Syn, Synonymous; Non-syn, Non-synonymous; n/a, Not Applicable.

**Ortholog Reference ID**	**Syn Count**	**Non-syn Count**	**Non-syn Nucleotide position**	**Non-syn Nucleotide Change**	**Non-syn Amino Acid Change**
E.pallida_nvec141000032_1	2	1	47	G/A	R/K
E.pallida_nvec141000032_2	2	0	n/a	n/a	n/a
E.pallida_nvec141000032_3	2	0	n/a	n/a	n/a
E.pallida_nvec3000224	1	0	n/a	n/a	n/a
E.pallida_nvec42000019_1	5	2	223; 369	A/T; A/T	T/S; E/D
E.pallida_nvec42000019_2	3	1	242	G/A	S/N
E.pallida_nvec7000121	5	0	n/a	n/a	n/a
E.pallida_tadh6000210_1	0	2	7;38	A/C; C/T	S/R; A/V
E.pallida_tadh6000210_2	4	0	n/a	n/a	n/a

Supplementary Table 6: Intron-exon structure analysis of nine *E. pallida* globin genes. Gene, exon and intron lengths are given as nucleotide counts. N/A used to identify introns with large blocks of ambiguous nucleotides, thus length of intron was not reported. Abbreviations: forward, F; reverse, R.

**Ortholog Reference ID**	**Scaffold Reference**	**Alignment Direction**	**Gene Length**	**Exon 1**	**Intron 1**	**Exon 2**	**Intron 2**	**Exon 3**
E.pallida_nvec141000032_1	18385412	F	516	143	219	226	795	147
E.pallida_nvec141000032_2	18385412	F	513	146	1366	226	537	141
E.pallida_nvec141000032_3	18385051	F	558	159	1437	225	343	174
E.pallida_nvec3000224	18385051	R	543	173	911	226	146	144
E.pallida_nvec42000019_1	18387879	F	546	173	2619	217	N/A	156
E.pallida_nvec42000019_2	18387879	F	534	170	N/A	217	N/A	147
E.pallida_nvec7000121	18385098	R	528	155	980	226	470	147
E.pallida_tadh6000210_1	18388191	F	498	161	1076	208	719	129
E.pallida_tadh6000210_2	18385444	R	486	146	N/A	205	260	135

Supplementary Figure 1: Cladogram of phylogenetic relationships for early diverging species, derived from mitochondrial (Rodríguez et al. 2014) and genomic (Zapata et al. 2015) genes. Candidate globin gene copy number in brackets after species name. Abbreviations: O, Order; C, Class.

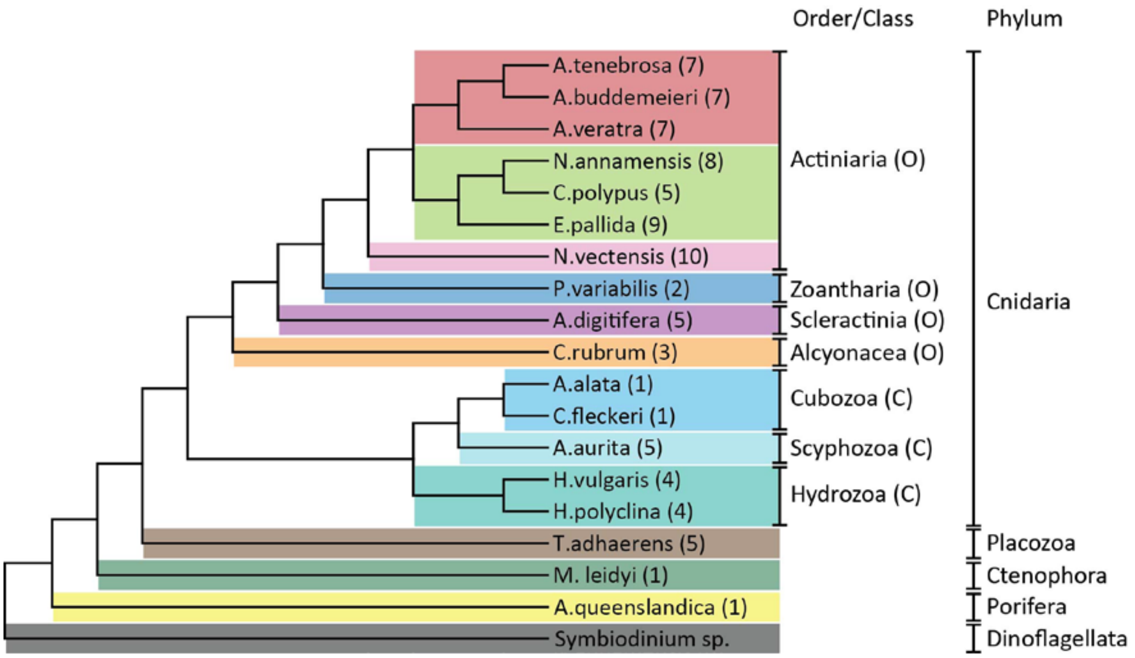

Supplementary Figure 2: Maximum Likelihood (ML) phylogenetic tree of identified candidate globin genes in transcriptomes of cnidarian species, with ML bootstrap and Bayesian posterior probability support values. Model and non-model representations of vertebrate globin genes, cnidarian classes Anthozoa, Cubozoa, Hydrozoa and Scyphozoa, with *S. minutum* as the outgroup. Support values shown as ML bootstrap support (0-100)/Bayesian posterior probabilities (0-1.0). Bootstrap values < 50 and posterior probabilities < 0.5 shown with a ~ symbol and nodes not identical between each method shown with a - symbol.

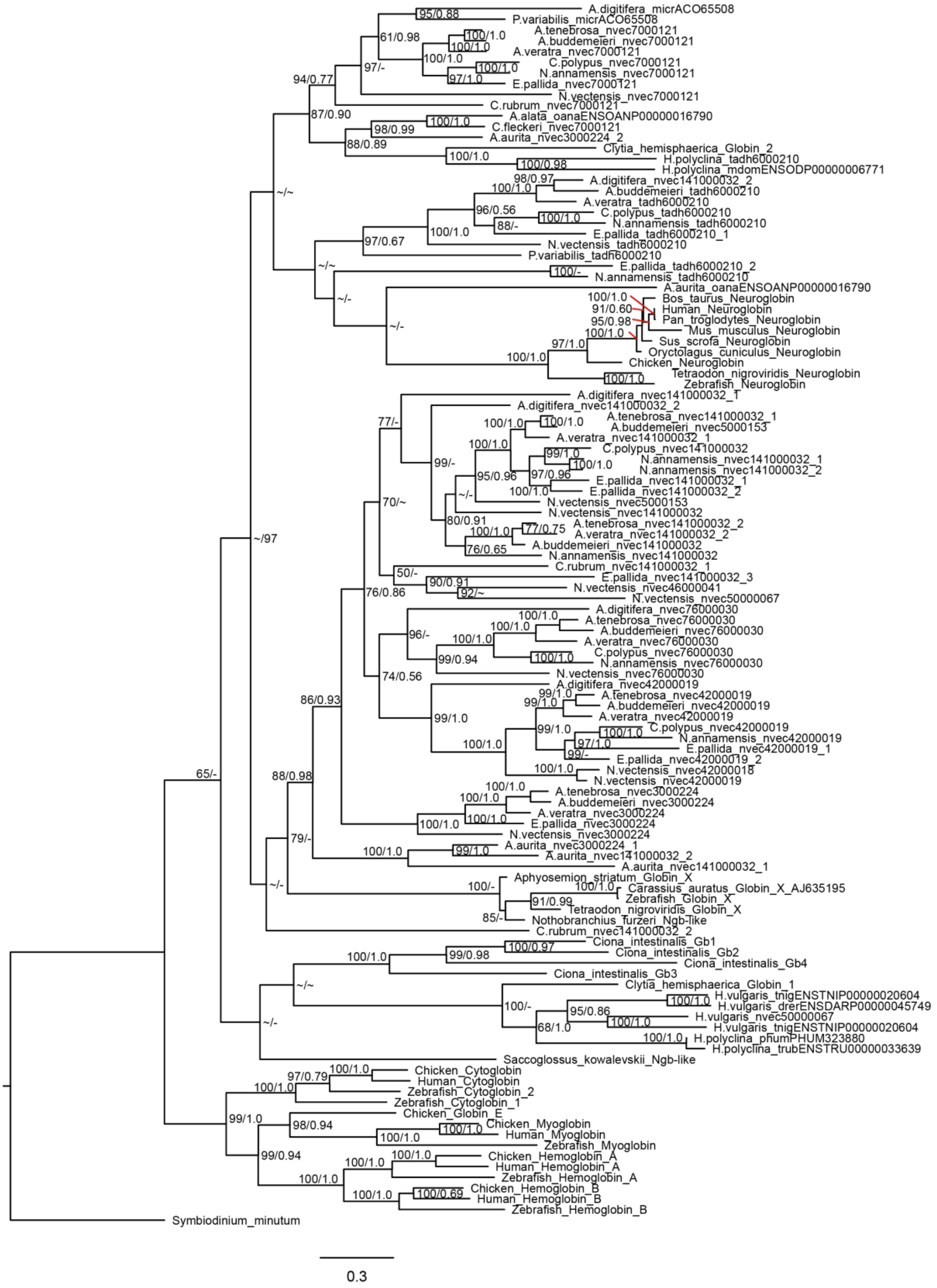

Supplement: Supplementary Data [file evy128_supp.pdf]
